# Topical Delivery of Ceramide by Oil-in-Water Nanoemulsion to Retain Epidermal Moisture Content in Dermatitis

**DOI:** 10.3390/biom15050608

**Published:** 2025-04-22

**Authors:** Yu Zhou, Lichun Wu, Yi Zhang, Jia Hu, Jannatul Fardous, Yasuhiro Ikegami, Hiroyuki Ijima

**Affiliations:** 1 Department of Chemical Engineering, Faculty of Engineering, Graduate School, Kyushu University, 744 Motooka, Nishi-ku, Fukuoka 819-0395, Japan; zhou.yu.222@s.kyushu-u.ac.jp (Y.Z.); wu.lichun.820@s.kyushu-u.ac.jp (L.W.); hu.jia.511@s.kyushu-u.ac.jp (J.H.); yikegami@chem-eng.kyushu-u.ac.jp (Y.I.); 2 Institute for Materials Chemistry and Engineering, Kyushu University, 744 Motooka, Nishi-ku, Fukuoka 819-0395, Japan; zhang_yi@ms.ifoc.kyushu-u.ac.jp; 3 Department of Pharmacy, Faculty of Science, Comilla University, Cumilla 3506, Bangladesh; fardousj11@cou.ac.bd

**Keywords:** transdermal delivery, permeability, ceramide, skin barrier function

## Abstract

External environmental stressors and internal physiological changes frequently compromise the skin barrier, resulting in conditions such as dermatitis and dehydration. A key underlying factor is the depletion of ceramides, essential lipids in the stratum corneum that maintain skin integrity. Although topical ceramide supplementation is effective for barrier repair, its clinical application is limited by poor solubility and low skin permeability. To overcome these challenges, this study developed an oil-in-water nanoemulsion (O/W-NE) using ultrasonic emulsification for the efficient transdermal delivery of ceramide C2. Octyldodecanol was selected as the oil phase to enhance ceramide solubility, while glycerin was incorporated to increase aqueous phase viscosity, reduce particle size, and function as a biocompatible penetration enhancer. The optimized nanoemulsion achieved a particle size of 112.5 nm and an encapsulation efficiency of 85%. Its performance was evaluated via in vitro release, ex vivo skin permeation, and in vivo biocompatibility studies. Mechanistic investigations revealed that both particle size and glycerin concentration significantly influenced ceramide penetration into the epidermis and dermis. Additionally, the nanoemulsion exhibited moisturizing and barrier-repair effects in a damaged skin model. Overall, this O/W-NE offers a stable, non-invasive strategy for enhancing ceramide delivery and restoring skin barrier function.

## 1. Introduction

The skin, which is the largest organ of the human body, functions as the primary barrier against external environmental factors, including UV radiation, pollution, and harsh weather conditions. These external aggressors, along with internal physiological changes, can compromise the skin barrier dysfunction, leading to dermatological conditions such as atopic dermatitis, eczema, and dehydration [1,2]. One of the critical components in maintaining the integrity of the skin barrier is ceramide, a type of sphingolipid, which is vital in retaining moisture and ensuring skin health. Topical ceramide delivery has emerged as a promising therapeutic strategy for restoring the skin barrier in individuals with compromised skin [3].

Ceramides are naturally occurring lipids found within the stratum corneum, the outermost layer of the skin. These molecules are essential for maintaining the skin’s barrier function by forming a structured lamellar matrix, which regulates water retention and prevents trans-epidermal water loss (TEWL) [4,5]. Ceramides, such as ceramide NP or ceramide EOS, differ in the length of the fatty acid chain and type of head group they carry, which affects how they behave in the skin formulation. For example, ceramide C2 is a relatively simple ceramide with a fatty acid chain and a sphingosine skeleton, making it ideal for skin barrier repair and hydration applications [6]. Reduced ceramide levels in the skin have been associated with various dermatological disorders, such as psoriasis and atopic dermatitis [7]. Replenishing ceramide levels, it is possible to enhance the skin’s ability to retain moisture and strengthen its defense against irritants, allergens, and pathogens, thus promoting healthier skin [8]. However, the topical application of ceramides presents certain challenges [9]. One of the primary limitations hindering the effective topical delivery of ceramides is their low solubility and limited permeability through the stratum corneum. These properties hinder ceramides from reaching deeper layers of the epidermis, where they are required to restore barrier function [10]. Several commercial lotions and creams (e.g., Eucerin Smoothing Repair Dry Skin Lotion and CeraVe Moisturizing Lotion) contain ceramides 1 and 3. Although these formulations are designed to help hydrate the skin and restore the skin barrier, there are challenges related to their permeability and efficacy. Studies show that ceramides in these products can improve skin hydration and reduce TEWL, and their effectiveness is boosted by the inclusion of penetration enhancers such as glycyrrhetinic acid or ethoxydiglycol [11]. However, studies such as those by Zhang et al. show that suspended ceramides fail to effectively penetrate the stratum corneum [12].

To overcome the limitations of conventional formulations, several advanced delivery systems such as liposome, nanoparticles, and emulsions have been developed. Ceramide-based liposomes have been developed to mimic the natural lipid bilayers of the skin. These liposomes, typically composed of ceramides, cholesterol, and fatty acids, are intended to restore the lipid barrier in damaged skin. Their high membrane fluidity and fusion activity with the stratum corneum make them effective carriers [13]. Despite their potential, the topical application of ceramides presents challenges, such as their content of organic solvents unsuitable for facial skin and sensitive areas [14]. Nanoparticles and microparticles aim to deliver ceramides in a controlled and targeted manner. While nanoparticles can achieve 60% penetration, their main advantage lies in controlled release rather than immediate penetration. Emulsions have emerged as effective platforms for enhancing ceramide delivery. These formulations improve penetration through the stratum corneum primarily due to their small droplet size and high surfactant content, which increase the solubility of ceramides, thereby improving their thermodynamic activity. Nanoemulsions showed a higher penetration rate of approximately 92% compared to other nanocarriers [15]. They are colloidal dispersions of oil droplets in water, typically with a droplet size range of 20–200 nm. Owing to their small particle size and large surface area, nanoemulsions exhibit enhanced solubility for lipophilic drugs, increased permeability, and improved bioavailability [16,17].

In particular, O/W-NEs can increase ceramides’ solubility through the inner oil phase and feel good when used. The outer water phase enhances hydration and barrier repair, making it a more comprehensive solution for treating skin diseases related to the epidermal barrier, unlike other carriers that may only focus on one aspect. At the same time, droplet size is critical in the efficacy of these systems, as smaller droplets can penetrate deeper into the skin [18,19]. Penetration into deeper skin layers is facilitated by improving the formulation [20]. For transdermal drug delivery, the nanoemulsion system enhances skin permeability without damaging the skin’s natural barrier, making it a suitable carrier for delivering ceramides. In summary, O/W-NEs provide a safe and non-invasive method to deliver active compounds to deeper skin layers [21]. They can deliver more active ingredients while maintaining skin moisture. This provides them with considerable advantages in the treatment of conditions such as eczema and psoriasis that are associated with a compromised skin barrier [22].

This study aims to develop and optimize an O/W-NE system for the effective transdermal delivery of ceramide C2, targeting skin disorders associated with barrier dysfunction. The novelty of this work lies in its dual-functional design: glycerin is incorporated not only to regulate the aqueous phase viscosity and optimize particle size, but also to act as a safe and effective penetration enhancer. This promotes both hydration and enhanced delivery of ceramide into the epidermis and dermis. More importantly, this study holds significant value in advancing topical ceramide therapy. Despite the recognized role of ceramides in barrier maintenance, their poor solubility and permeability limit clinical efficacy. By addressing these challenges through a stable, non-invasive, and biocompatible delivery platform, this work contributes to improving treatment strategies for chronic skin conditions such as atopic dermatitis and eczema. Furthermore, by elucidating the role of formulation parameters in enhancing lipid-based drug delivery, this study provides mechanistic insights that can inform the design of future transdermal systems. Overall, this research bridges critical gaps in the topical application of ceramides and supports the development of clinically relevant, patient-friendly dermatological formulations.

## 2. Materials and Methods

### 2.1. Materials

#### 2.1.1. Reagents

Ceramide C2 (Fujifilm Wako Pure Chemical Corp., Osaka, Japan) was used as a hydrophobic drug with poor water solubility (lower than 0.001% in water) [23]. Purified water and glycerin (Fujifilm Wako Pure Chemical Corp., Osaka, Japan) were used as the continuous phase (aqueous phase) and octyldodecanol (Tokyo Chemical Industry Co., Ltd., Saitama, Japan) as the dispersed oil phase. Tween 80 (Tokyo Chemical Industry Co., Ltd., Saitama, Japan), a surfactant with an HLB value of 15.0, was used to form the O/W-NE. NBD-Ceramide (Fujifilm Wako Pure Chemical Corp., Osaka, Japan) is a fluorescent derivative of ceramide used to evaluate the O/W-NE-mediated distribution of ceramide in the skin. All the materials used in nanoemulsion preparation and related experiments were of analytical grade.

#### 2.1.2. Animals

ICR mice (Institute of Cancer Research mice, male, six weeks old, Japan SLC Inc., Shizuoka, Japan; average weight: 28 ± 5 g) and HR-1 (Hairless-1 mice, male, six weeks old, Japan SLC Inc., Shizuoka, Japan; average weight: 25 ± 5 g) were housed in cages (two mice per cage) in a temperature-controlled environment (25 ± 1 °C) with a 12 h light/dark cycle. They were given unrestricted access to a standard diet. Anesthesia was induced using inhalational isoflurane for all surgical procedures to minimize pain and distress. The experimental procedures followed protocols sanctioned by the Ethics Committee on Animal Experiments of Kyushu University (protocol code A23-478-0, approval date 21 December 2023).

### 2.2. Development of O/W-NE

#### 2.2.1. Preparation of O/W-NE

Preparation conditions for O/W-NE and ceramide C2-loaded O/W-NE were investigated. Specifically, the aqueous phase was made by dissolving predetermined amounts of Tween 80 as a surfactant in the mixture of glycerin and deionized water at 70 °C in a water bath. The same experimental condition was maintained, with octyldodecanol in a separate tube serving as the oil phase. The oil phase was then added to the aqueous phase, followed by emulsification through ultrasonication (Branson Sonifier 250, Branson Ultrasonics Corp., Danbury, CT, USA) for 10 min with one pulse/0.7 s and 70% power amplitude. The temperature was maintained at 70 °C during emulsification using a water bath (THERMAX TM-1A, AS ONE Corp., Osaka, Japan). To prepare ceramide C2-loaded O/W-NEs, ceramide C2 was pre-dissolved in octyldodecanol to form the oil phase. The rest of the method was the same as that of the O/W-NE preparation.

#### 2.2.2. Optimization of Formulation

A stable O/W-NE formulation requires a suitable surfactant concentration and glycerin content, the optimal volume of oil and aqueous phase, and the optimum ultrasonic time and temperature during preparation [24]. The formulation of the nanoemulsion was optimized according to Table 1. Specifically, oil and aqueous phases at volume ratios of 1:9, 2:8, 3:7, and 4:6 were used to obtain nanosized particles. Similarly, the surfactant at a concentration range of 25–150 mg/mL in the aqueous phase was used to develop an O/W-NE formulation with the desired particle size and polydispersity index (PDI). For glycerin content in the aqueous phase, glycerin and deionized water at volume ratios of 1:7, 1:3, 1:1, 3:1, and 7:1 were used. In addition, an ultrasonic temperature from 25 °C to 80 °C and an ultrasonic time from 5 min to 30 min were used. A stable O/W-NE formulation was developed with an optimum concentration of each ingredient.

### 2.3. Characterization of O/W-NEs

#### 2.3.1. Measurement of Particle Size and PDI

The average particle size and its distribution characterized by the PDI of the O/W-NEs were measured using dynamic light scattering (DLS; Nano-SAQLA, Otsuka Electronics Co., Ltd., Osaka, Japan) with a laser scattering particle analyzer. The measurement was performed at 20 °C and at a scattering angle of 173°. O/W-NEs were diluted 600 times with purified water before measurement of particle size and PDI. Zeta potential of the prepared nanoemulsion was measured after 1000-fold dilution using a Malvern Zetasizer (Zetasizer Nano ZS, Malvern Panalytical Ltd., Malvern, UK).

#### 2.3.2. Solubility of Ceramide C2 in O/W-NE

The solubility of ceramide C2 was determined by adding 50 µg of ceramide C2 to O/W-NE and deionized water. After allowing the mixtures to settle, a microscope was used to examine the solutions. Solubility was confirmed when no crystalline structures, crystal needles, or fine strings were observed under the microscope.

#### 2.3.3. Morphology of Ceramide C2-Loaded O/W-NE

The morphology of nanoparticles was observed using transmission electron microscopy (TEM). Samples for TEM images were prepared by dilution of O/W-NE and ceramide C2-loaded O/W-NE (100 times), and 5 μL was placed on a carbon-coated copper grid, dried at 25 °C for 30 min, vacuumed for 24 h, and finally observed under TEM at 120 kV (JEM-2100, JEOL Ltd., Tokyo, Japan).

### 2.4. Encapsulation Efficiency (EE) of Ceramide C2-Loaded O/W-NE

EE is the percentage of drugs that are successfully entrapped in a nanoparticle. To evaluate the EE of ceramide C2 in O/W-NE, 1 mL of ceramide C2-loaded O/W-NE was centrifuged at 21,380× *g* for 30 min at 20 °C. Free ceramide C2 content was determined by measuring the non-incorporated drug present in a clear supernatant obtained through the separation of the aqueous phase [24].

To establish the linearity of the proposed method, a standard curve was constructed at six concentration levels within the range of 1–50 μg/mL. These ceramide C2 standard solutions were prepared using ethanol/Phosphate Buffered Saline (PBS) (3:7) as a solvent. Least square regression analysis was performed for the ceramide C2 standard solution absorbance data (y = 0.0216x + 0.0491, R^2^ = 0.9966). Beer’s law was obeyed in all spectrophotometric analyses. Ceramide C2 concentration in this solvent system was determined using a UV/VIS spectrophotometer (UV-2500PC, Shimadzu Corp., Kyoto, Japan) with detection at 207 nm. The EE of ceramide C2 into O/W-NE was calculated using Equation (1) [25,26]:

(1) $$EE\%=\frac{\left(Total\;drug\;added-Non\;incorporated\;drug\right)}{Total\;drug\;added}\times 100\%$$

### 2.5. Stability of O/W-NE

The physical stability of the O/W-NE was determined through the analysis of particle size and PDI. The NEs were stored at three different temperatures, as follows: 4 °C, 25 °C, and 37 °C for four months, and samples were subsequently tested for particle size and PDI at different time intervals.

### 2.6. In Vitro Drug Release from O/W-NE

Using Coumarin-6 as a model drug, the drug release in vitro from O/W-NE was studied. First, O/W-NE containing the fluorescence Coumarin-6 (Tokyo Chemical Industry Co., Ltd., Tokyo, Japan) was prepared, and the preparation method is shown in Section 2.2.1. Subsequently, O/W-NE was suspended in a release medium (Isopropyl myristate, Fujifilm Wako Pure Chemical Co., Osaka, Japan) at different temperatures (4 ± 0.5 °C, 37 ± 0.5 °C) and incubated under different conditions (static or continuous shaking (100 rpm)). At specified time intervals, the release medium was removed, centrifuged, and the fluorescence intensity of Coumarin-6 in the supernatant was measured using a fluorometer at excitation/emission wavelengths of 436 nm/525 nm. A standard curve of Coumarin-6 was constructed to calculate the percentage of drug release over time. The Coumarin-6 standard diagram was used to determine the percentage release of Coumarin-6 as a function of time, as follows (2) [27,28]:

(2) $$Drug\;released(\%)=\frac{The\;content\;of\;Coumarin-6\;at\;different\;time\;points}{The\;total\;content\;of\;Coumarin-6\;added}\times 100\%$$

The release kinetics were analyzed using four models: zero-order, first-order, Korsmeyer–Peppas, and Weibull models (3, 4, 5, 6 and 7). Details of these models can be found in previous studies [29].

Zero-order release model (3) [30,31]:

(3) $$Q_{t}=Q_{0}+k_{0}\cdot t,$$

*Q_t_* is the cumulative amount of drug released at time *t*, *Q*_0_ is the initial drug amount, and *k*_0_ is the zero-order release rate constant.

First-order release model (4) [30,32]:

(4) $$\frac{dC}{dt}=-k\cdot C,$$

After integration:

(5) $$Q_{t}=Q_{\infty }\cdot (1-e^{-k\cdot t}),$$

*C* is the drug concentration, *Q_∞_* is the total amount of drug released, and k is the first-order release rate constant.

Korsmeyer–Peppas model (6) [33,34,35]:

(6) $$\frac{Q_{t}}{Q_{\infty }}=k\cdot t^{n},$$

*Q_t_/Q_∞_* represents the fraction of drug released at time *t*, *k* is the release constant, and *n* is the diffusion exponent. The value of *n* indicates the mechanism: *n* = 0.5 corresponds to Fickian diffusion, 0.5 < *n* < 1.0 suggests anomalous (non-Fickian) transport, and *n* = 1.0 represents zero-order release [36].

Weibull model (7) [37]:

(7) $$F\left(t\right)=1-e^{{-\left(\frac{t}{T}\right)}^{b}},$$

*F*(*t*) is the fraction of drug released at time t, *T* is the scale parameter (time constant), and *b* is the shape parameter. This model accommodates single and multi-mechanistic release behaviors [38,39].

### 2.7. Ex Vivo Permeability Study

#### 2.7.1. Permeability Analysis of O/W-NEs

The transdermal delivery mechanism of O/W-NEs was investigated by examining the effect of particle size and glycerin. The particle size was adjusted by varying the oil phase content, and the influence of glycerin was assessed by preparing nanoemulsions with and without glycerin. The permeability was analyzed through transdermal delivery experiments using the hydrophobic fluorescent dye Nile red.

Ex vivo skin permeability tests were performed using a Franz diffusion cell with ICR mice’s dorsal skin as the transdermal membrane. A total of seven experimental groups were designed (e.g., different particle size or glycerin content), with three mice per group (*n* = 3), resulting in the use of 21 mice. After euthanizing the mice, the dorsal hair was shaved, and the skin was carefully stripped. The dorsal skin was placed between the donor and receptor compartments of the Franz cell, with 1 mL PBS in the receptor compartment. The temperature was maintained at 32 ± 0.5 °C. After 24 h, the skin was retrieved, embedded in an optimal cutting temperature compound (Sakura Finetek USA, Inc., Torrance, CA, USA), frozen at −80 °C, and sectioned into 20 µm slices. Fluorescence distribution was observed using confocal laser scanning microscopy (CLSM) (FV1000; Olympus, Tokyo, Japan), and fluorescence intensity in the skin was measured using ImageJ software (version 1.54f, National Institutes of Health, Bethesda, MD, USA).

#### 2.7.2. Penetration of NBD-Ceramide-Loaded O/W-NE

The transdermal permeability of NBD-ceramide-loaded O/W-NE was evaluated using NBD-ceramide as a model drug incorporated into the inner oil phase. ICR mice were divided into three groups: control, NBD-ceramide in octyldodecanol, and NBD-ceramide-loaded O/W-NE, with 2 mice per group (*n* = 2), resulting in the use of 6 mice. Distribution and fluorescence intensity of NBD-ceramide were observed using CLSM (485/530 nm).

### 2.8. Skin Surface Irritation Test In Vivo

To assess the skin safety of the O/W-NE, the formulations were applied to the dorsal skin of ICR mice. A total of three experimental groups were designed (e.g., control (no treatment), O/W-NE, CER-NE), with 2 mice per group (*n* = 2), resulting in the use of 6 mice. After 24 h, the skin was retrieved, fixed in neutral formalin, and subjected to hematoxylin and eosin (H&E) staining. The skin integrity was observed under a microscope to evaluate potential damage.

### 2.9. Efficacy Evaluation of Ceramide C2-Loaded O/W-NE In Vivo

Ceramide C2-loaded O/W-NE was prepared as described in Section 2.2.1. The formulation was applied to the dorsal skin of HR-1 mice in a skin injury model. A total of three experimental groups were designed, with 2 mice per group (*n* = 2), resulting in the use of 6 mice. Sterilized gauze (3 cm × 2 cm) soaked in 250 µL of 5 mg/mL antibacterial agent (Finibax for Intravenous Drip Infusion, Shionogi & Co., Ltd., Osaka, Japan) and 1 mL solution (ceramide C2-loaded O/W-NE or PBS) were placed on the skin and fixed with surgical tape. The Ceramide C2-loaded O/W-NE or PBS, gauze, and tape were changed daily for five days. Skin moisture was measured daily, and skin hydration capacity was assessed using a skin evaporative water logger (SR-101, LOZENSTAR Co., Ltd., Osaka, Japan). The ceramide C2-loaded O/W-NE group was compared to the control group (PBS).

### 2.10. Statistical Analysis

All test results are presented as mean ± standard deviation (S.D.). Statistical significance was determined using two-way ANOVA, followed by Tukey’s Honest significant difference (HSD) test for multiple comparisons.

## 3. Results and Discussion

### 3.1. Development of Nanoemulsion Formulation

**Figure 1:**
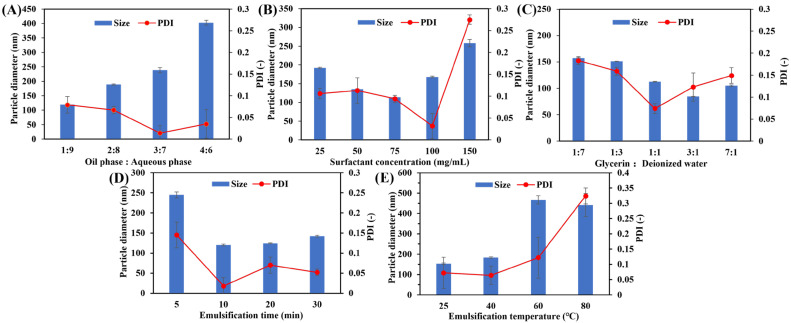
Particle size and PDI of O/W-NEs change with the formulation. (**A**) Oil–aqueous phase volume ratio, (**B**) surfactant concentration, (**C**) glycerin-deionized water volume ratio in aqueous phase, (**D**) emulsification time, (**E**) emulsification temperature. (*n* = 3, Bars: S.D.).

The particle size and PDI of O/W-NEs are influenced by various relevant factors (Figure 1). As shown in Figure 1A–C, increasing the aqueous phase volume and maintaining moderate levels of surfactant or glycerin reduces particle size and PDI. This can be attributed to a better dispersion of the oil phase within the continuous aqueous phase, reducing the likelihood of droplet coalescence. However, excessive surfactant or glycerin led to unfavorable outcomes, such as increased particle size and instability, possibly due to micelle saturation or phase separation during emulsification [17]. Glycerin, as a multifunctional component, plays a dual role. It increases the viscosity of the aqueous phase, thereby reducing droplet collision and coalescence during emulsification, which contributes to smaller particle sizes and narrower distribution [40]. At appropriate concentrations, glycerin also acts as a safe penetration enhancer. Nevertheless, when used in excess, it may disrupt nanoemulsion stability by altering the phase balance or leading to phase separation [41,42].

The emulsification process parameters, including time and temperature, also had a pronounced impact on droplet size distribution. Ultrasonication for 5–30 min showed that increasing the emulsification time initially reduced particle size due to more efficient droplet disruption and surfactant dispersion. However, after 10 min, further time extension had minimal effects (Figure 1D), suggesting a saturation point in energy input efficiency [43]. Thus, 10 min was selected as the optimal time, balancing particle size reduction and processing efficiency. As the temperature increased to 80 °C, both particle size and PDI increased (Figure 1E). This phenomenon aligns with previous findings that the performance of nonionic surfactants, such as Tween 80, is highly temperature-dependent [44]. Elevated temperatures may dehydrate the polyoxyethylene head groups of Tween 80, reducing its hydrophilicity and interfacial stability, thus promoting desorption from the oil–water interface and leading to droplet aggregation [45]. Therefore, maintaining a moderate emulsification time and temperature is critical for achieving optimal droplet characteristics and formulation stability.

According to previous studies, nanoemulsions with smaller particle sizes exhibit improved transdermal delivery. Taking drug loading into consideration, a particle size of approximately 100 nm with a monodisperse distribution (PDI < 0.3) was selected [18,46]. Through a single-variable design approach, the optimized formulation of O/W nanoemulsion consisted of an oil–aqueous phase ratio of 1:9, 75 mg/mL surfactant, glycerin-deionized water at 1:1 to form the aqueous phase, and ultrasonic emulsification at 25 °C for 10 min to prepare the nanoemulsions.

### 3.2. Characterization and Morphology of O/W-NE and Ceramide C2-Loaded O/W-NE

**Figure 2:**
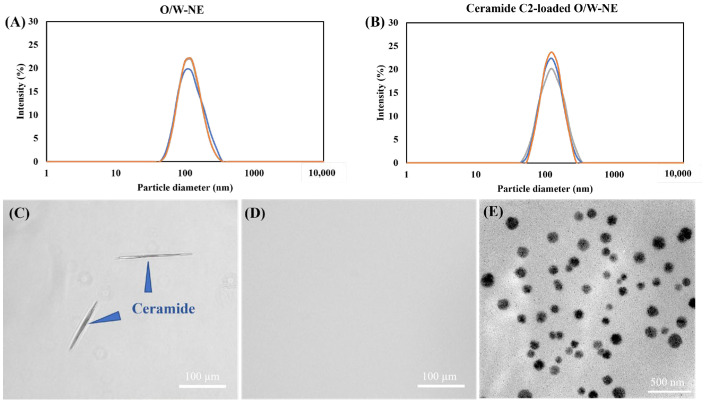
Characterization of O/W-NEs by (**A**) particle size and distribution of O/W-NE, (**B**) particle size and distribution of ceramide C2-loaded O/W-NE, (The orange, blue, and grey lines represent three consecutive DLS measurements of the same sample.) (**C**) micrograph of ceramide C2 in water (Bar: 100 µm), (**D**) micrograph of ceramide C2-loaded O/W-NE (Bar: 100 µm), (**E**) TEM micrograph of ceramide C2-loaded O/W-NE (Bar: 500 nm).

The results of particle size and the distribution of O/W-NEs are shown in Figure 2A,B. Particle size analysis by DLS shows that the O/W-NE had nanosized particles of 112.5 nm in diameter. The PDI value of 0.094 indicates homogenous, uniformly sized, spherical vesicles. Similarly, no notable change was observed in any of these parameters in the presence of the hydrophobic drug ceramide C2. Compared to previously reported ceramide-loaded nanoemulsions, which showed particle sizes ranging from 150 to 220 nm, the smaller particle size in our formulation may offer potential advantages for transdermal delivery [15,47]. Smaller droplets provide a larger surface area, enhancing skin contact and promoting diffusion across the stratum corneum. Their nano-scale size allows easier passage through the intercellular lipid channels of the stratum corneum and facilitates closer interaction with skin components, which enhances permeation [48,49]. The prepared ceramide C2-loaded O/W-NE and ceramide aqueous solution were observed under a microscope. As shown in Figure 2C,D, in water solutions, ceramide crystals can be clearly observed. However, encapsulating ceramide in O/W-NE improves their solubility. O/W-NE is expected to be a carrier for transdermal delivery of ceramide. The high specific surface area of the nanoemulsion particles enhances the dissolution and diffusion of ceramide, thereby improving its solubility in the nanoemulsion [50].

The mean values of the physicochemical properties of the O/W-NEs are shown in Table 2. After the nanoemulsion contained ceramide C2, the nanoparticles’ particle diameter and Zeta potential barely changed, indicating that ceramide was encapsulated inside the nanoemulsion instead of existing at the interface or surface. TEM observed the morphology and size of nanoparticles in O/W-NE. Figure 2E shows that the ceramide C2-loaded O/W-NE was in the form of uniformly distributed round spheres. The TEM image results are roughly consistent with the DLS measurements. This is in line with previous studies, which report that while DLS tends to show slightly larger sizes due to hydrodynamic radius measurement, TEM provides direct visualization of dehydrated particle cores [51].

### 3.3. EE of Ceramide C2-Loaded O/W-NE

To determine the concentration of ceramide C2, it is necessary to obtain its standard curve. According to previous studies, there is a linear relationship between the concentration and absorbance of ceramide C2. The EE of the nanoemulsion was determined by quantifying the free ceramide in the nanoemulsion. Using equation (1) to calculate, the EE of ceramide is 85%, within this acceptable range, and supports adequate drug loading [52,53]. This result aligns with the findings of Tessema et al., who reported an EE of 85% using starch-based nanoparticles, and is comparable to that of Gaur et al., who achieved 90% EE in solid lipid nanoparticles containing ceramide 2 [15,54]. Together with the smaller particle size (113 nm), our formulation demonstrates a favorable balance between encapsulation and delivery potential. These findings suggest that the nanoemulsion structure effectively improves the solubility and encapsulation of hydrophobic compounds such as ceramide. This is consistent with the result in Figure 2C,D. This finding highlights the role of the O/W-NE system in optimizing ceramide delivery, despite the inherent challenges posed by ceramide’s low water solubility.

### 3.4. Long-Term Stability of O/W-NEs Under Different Temperature Conditions

**Figure 3:**
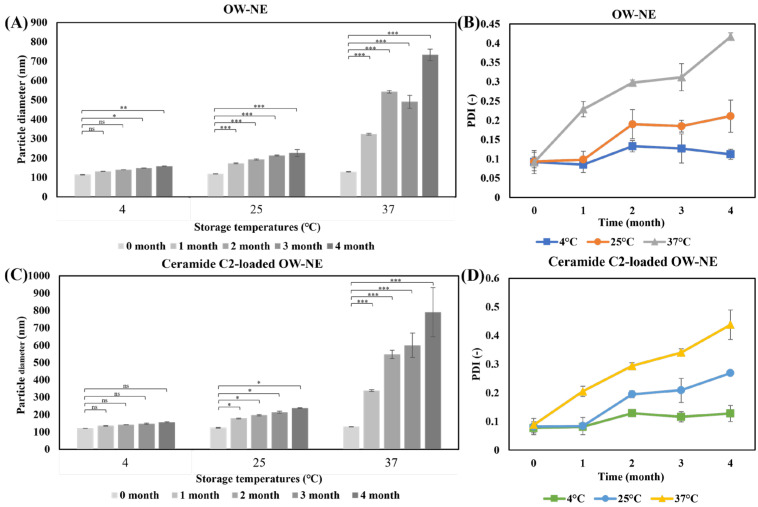
Time-dependent stability of O/W-NEs under different storage temperatures. (**A**) Particle size of O/W-NE, (**B**) dispersion of O/W-NE, (**C**) particle size of ceramide C2-loaded O/W-NE, (**D**) dispersion of ceramide C2-loaded O/W-NE. (Bars: S.D., *n* = 3, ns: *p* > 0.05, *: *p* < 0.05, **: *p* < 0.01, ***: *p* < 0.001).

A four-month stability study of O/W-NEs under different storage conditions demonstrated that the nanoemulsion remained stable at 4 °C and 25 °C, but exhibited limited stability at 37 °C (Figure 3). These findings are consistent with the general understanding that lower temperatures inhibit molecular motion, thereby reducing nanoparticle aggregation and preserving emulsion stability over extended periods [55,56]. As the temperature increases, the reduction in interfacial tension, accelerated coalescence of oil droplets, and decreased emulsifier stability lead to an increase in particle size and PDI [42]. At 4 °C, the particle size (114–158 nm) and PDI (0.092–0.112) remain unchanged for a prolonged period. Statistical analysis revealed no significant differences (*p* > 0.05) in particle size and PDI over the two-month period, indicating high stability under refrigeration, suitable for long-term storage. At 25 °C, the particle size increased to approximately 200 nm after four months, yet remained within the range suitable for effective transdermal delivery [57]. This highlights the potential for 25 °C storage in practical applications, with minimal compromise to functionality. In contrast, the 37 °C conditions showed a relative increase, with approximately one month of stability. Instability may have been due to nanoparticle flocculation caused by structural degradation at elevated temperatures [58]. This temperature-dependent instability may facilitate the release of encapsulated ceramides, supporting their intended function upon application, as previously reported for similar delivery systems [59]. Notably, the incorporation of ceramide C2 did not significantly alter the stability profile, confirming the robustness of the nanoemulsion formulation (Figure 3C,D).

In summary, refrigeration is the ideal condition for long-term storage of O/W-NEs to maintain their application. While O/W-NEs gradually degrade at 37 °C over time, this temperature-responsive behavior suggests a potential mechanism for the functional release of encapsulated ceramide. The combination of storage stability and thermal responsiveness indicates that O/W-NEs may be a promising platform for transdermal delivery systems, though further studies under application-relevant conditions are needed.

### 3.5. In Vitro Drug Release

**Figure 4:**
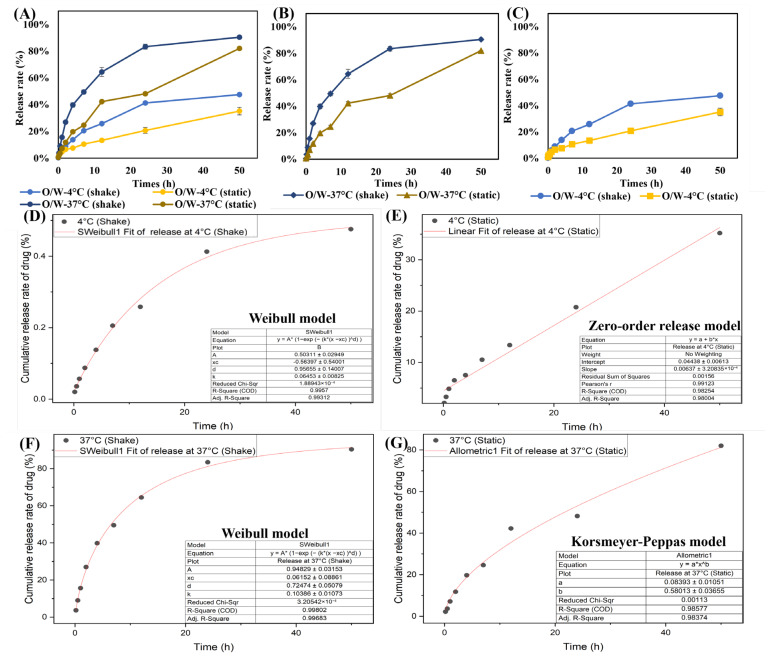
Drug release from O/W-NEs and kinetic evaluation of release profile. (**A**) Drug release from O/W-NEs under different conditions, (**B**) drug release from O/W-NEs at 37 °C under different conditions, (**C**) drug release from O/W-NEs at 4 °C under different conditions, (**D**) drug release at 4 °C (shake), data fitted to Weibull model, (**E**) drug release at 4 °C (static), data fitted to zero-order release model, (**F**) drug release at 37 °C (shake), data fitted to Weibull model, (**G**) drug release at 37 °C (static), data fitted to Korsmeyer–Peppas model. (Bars: S.D., *n* = 3).

The cumulative release of Coumarin-6 from the oil droplets of O/W-NE under different conditions is shown in Figure 4A. Coumarin-6 was selected as a model hydrophobic compound to evaluate the release behavior from O/W-NEs due to its strong fluorescence and ease of quantification [60]. However, given the structural and solubility differences from ceramide, the results are intended as a general reference rather than a direct representation of ceramide-release kinetics.

To investigate how environmental factors influence drug release, two temperatures were selected: 4 °C to represent a low-temperature storage condition, and 37 °C to simulate physiological environments commonly used in in vitro studies. This design allows evaluation of both nanoemulsion stability and thermal responsiveness. It is evident that temperature and agitation considerably influence the drug release profile. Drug release at 37 °C is widely adopted to simulate the environment in the body and represents the release behavior of drugs at physiological temperatures. An increase in temperature may enhance molecular thermal motion, leading to the rupture of the nanoemulsion or disassembly of its structure. Additionally, higher temperatures increase solubility and decrease the viscosity of the release medium, resulting in a generally accelerated release rate at 37 °C compared to 4 °C. This difference can reflect the rate at which the drug or active ingredient is released in the body’s environment. In Figure 4B,C, the shaking accelerates nanoemulsion movement, causing structural instability and potential rupture of oil droplets. Under static conditions, drug release is primarily limited to the surface of the nanoemulsion in direct contact with the release medium. In contrast, shaking induces liquid flow and convection, continuously renewing the solution environment around the particles, thereby enhancing release efficiency. Meanwhile, the low-temperature and static environment helps maintain the structural integrity of the carrier, inhibiting droplet rupture or carrier degradation, and leads to a more stable and slower release profile. If the temperature or shaking considerably speeds up the release rate, the zero-order or diffusion-controlled model may be changed to a burst-release model [61].

The release profiles of the O/W-NE under various conditions reveal distinct mechanisms driven by temperature and environmental dynamics. Drug-release kinetics were analyzed using four models, revealing notable differences across conditions. By fitting the release curves to various models (Figure 4D–G), it was determined that the drug release under 4 °C (shaking) and 37 °C (shaking) conditions followed the Weibull release model. Under 4 °C (static) conditions, the release was best described by the zero-order model, while at 37 °C (static), the Korsmeyer–Peppas model provided the best fit.

These results indicate that temperature and agitation significantly influence the drug-release mechanisms of O/W-NE. The applicability of the Weibull model under shaking conditions suggests that complex multi-mechanistic processes govern the release, likely involving diffusion, dissolution, and interfacial mass transfer. The zero-order release profile observed at 4 °C (static) implies a constant release rate, possibly due to reduced kinetic activity at low temperatures, which limits diffusion and dissolution, allowing the release process to be controlled by the structural characteristics of the carrier. At 37 °C (static), the Korsmeyer–Peppas model (with b values between 0.5 and 1) suggests a non-Fickian diffusion mechanism, possibly involving swelling, erosion, or other complex processes. The stability and release of nanoemulsions are influenced by formulation parameters, such as preparation methods, emulsifier types, and environmental conditions, all of which impact the drug-release mechanism and rate. Therefore, considering these factors is crucial when designing O/W-NE for effective drug delivery.

### 3.6. Permeability Analysis of O/W-NE

**Figure 5:**
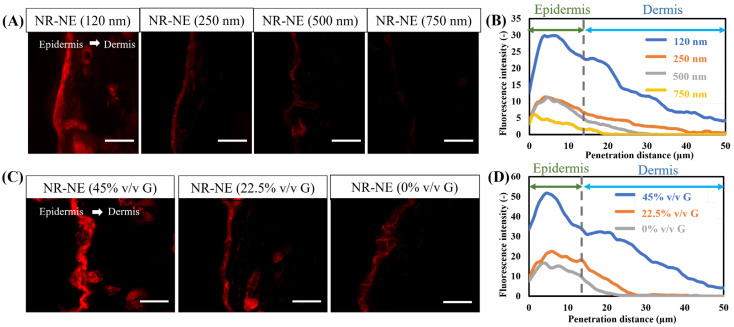
Ex vivo permeability. (**A**) Permeability of Nile red-loaded O/W-NE with different particle sizes, (**B**) quantitative analysis curve of fluorescence intensity and penetration distance for (**A**), (**C**) permeability of Nile red-loaded O/W-NE with different content glycerin, (**D**) quantitative analysis curve of fluorescence intensity and penetration distance for (**C**), (Bars: 100 µm, *n* = 3).

The results of the ex vivo transdermal experiments with different O/W-NEs are shown in Figure 5, indicating that both particle size and glycerin content significantly influence transdermal performance.

First, the results demonstrate that particle size has a substantial effect on transdermal penetration. A series of O/W-NEs with varying particle sizes were prepared by altering the oil phase content. In Figure 5A, the ex vivo transdermal experiment results reveal that O/W-NEs with smaller particle sizes markedly enhance the penetration depth and fluorescence intensity of Nile red compared to formulations with larger particle sizes (Figure 5B). This indicates that smaller particle sizes improve transdermal performance. However, it is important to note that “smaller is not always better.” Extremely small particles may reduce drug-loading efficiency and compromise formulation stability, resulting in lower drug encapsulation and accelerated release. Literature reports suggest that particle sizes of approximately 100 nm offer better transdermal performance and drug-loading efficiency, but further optimization is needed to achieve maximum therapeutic efficacy and enhanced transdermal delivery [18,20,62,63,64,65].

Second, in Figure 5C, the transdermal experiments using O/W-NEs with varying glycerin content demonstrate that glycerin content significantly influences skin permeability. As glycerin content decreases, the fluorescence intensity in the skin and penetration distance decreases (Figure 5D). This suggests that glycerin enhances the transdermal delivery of O/W-NEs. Glycerin, as an effective moisturizer, promotes skin hydration, temporarily reducing the tightness of the skin barrier and allowing for better penetration of drugs and active ingredients. Moreover, glycerin softens the stratum corneum, increases its fluidity, and widens the intercellular space, facilitating the penetration of drug molecules into deeper tissues [65,66].

These findings emphasize the importance of considering factors such as particle size and glycerin content when designing O/W-NE transdermal delivery systems in order to achieve optimal transdermal efficacy and therapeutic outcomes. In summary, O/W-NEs have proven to be a promising and advanced drug delivery system, not only due to their advantages such as ease of preparation, cost-effectiveness, stability, etc. The O/W-NEs can bypass the skin barrier and deliver hydrophobic drugs to the epidermis and dermis, and even into the systemic circulation, which is notable. Based on previous studies, several mechanisms for O/W-NE transdermal transport have been proposed [18,67,68]. These are divided into the following categories: (a) Disruption of the stratum corneum lipid bilayer, (b) enhancement of transdermal permeation through oil droplet nano-sizing, (c) binding of positively charged NE to negatively charged skin, (d) hydrating of skin and the dilation of the stratum corneum intercellular channels, and (e) changing of the permeation pathway of lipophilic permeants to follicular delivery [18]. In this study, the mechanism of O/W-NE transdermal delivery was investigated from two main perspectives: the effect of nanoemulsion particle size and the role of glycerin.

### 3.7. In Vitro Permeability Study of Ceramide Mediated-Loaded O/W-NE

**Figure 6:**
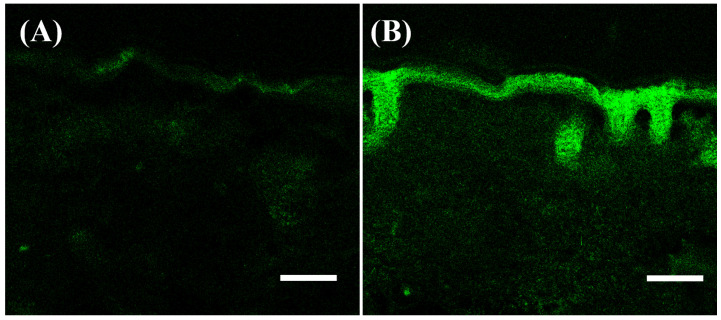
Ex vivo permeability of O/W-NE containing NBD-ceramide. (**A**) Permeability of NBD-ceramide in the oil phase, (**B**) permeability of NBD-ceramide-loaded O/W-NE. (Bars: 100 µm, *n* = 3).

NBD-ceramide, a fluorescent derivative of ceramide, was used to evaluate the O/W-NE-mediated distribution of ceramide in the skin. The results demonstrated significantly improved permeability of the O/W-NE formulation compared to the oil solution. As observed in Figure 6, treatment with NBD-ceramide oil showed only a minimal amount of fluorescence in the cuticle. However, the NBD-ceramide in the O/W-NE showed a large amount of NBD-ceramide enriched in the epidermis and edge of the dermis, indicating deeper O/W-NE-mediated delivery and drug penetration through the cuticle and especially through the hair follicle. In the structure of the skin, the stratum corneum is lipophilic, while the underlying skin is hydrophilic. Therefore, the oil solution of Nile red can only exist in the stratum corneum and it is difficult for it to enter the skin. However, the O/W nanocarrier can enhance skin hydration, improve the mobility of the stratum corneum, and make the cell space larger [69]. Additionally, it has a small particle size (~100 nm) that allows it to pass through the intercellular spaces in the stratum corneum and into the epidermis [70]. The experimental results show that O/W-NE enhances the skin permeability of ceramide and can be delivered to the epidermis and the edge of the dermis, which is expected to treat skin surface diseases.

### 3.8. Non-Irritating Skin Effect of C2-Loaded O/W-NE In Vivo

**Figure 7:**
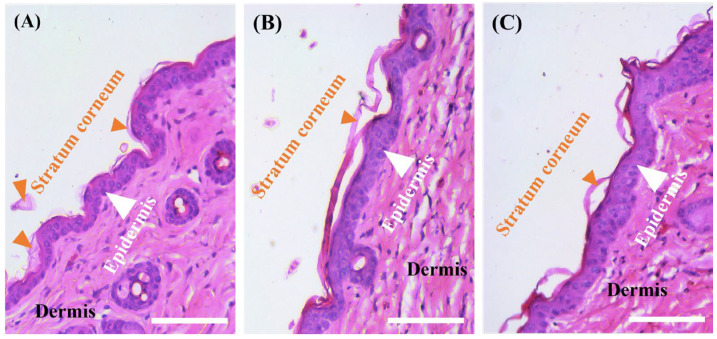
H&E staining after application of the O/W-NEs for 24 h. (**A**) No treatment (healthy skin), (**B**) treatment by O/W-NE, (**C**) treatment by ceramide C2-loaded O/W-NE. (Bars: 100 µm, *n* = 2).

Figure 7 presents the results of the skin irritation test following 24 h treatment with O/W-NE and ceramide C2-loaded O/W-NE formulations. Compared with the untreated group, no signs of erythema or edema were observed at the application sites after removal of the test materials. Skin sections were prepared and observed after H&E staining, and the results are shown in Figure 7. The images of treatment with O/W-NEs showed well-defined and adjacent margins between the stratum corneum, epidermis, and dermis. As illustrated in Figure 7B,C, the stratum corneum remained intact, no inflammatory cell infiltration was observed in the dermis, the skin appendages appeared normal, and collagen fiber bundles were densely arranged. There were no notable differences compared to the no-treatment group (healthy skin). This indicates that O/W-NE and ceramide C2-loaded O/W-NE cause no skin irritation.

### 3.9. Efficacy Evaluation of Ceramide C2-Loaded O/W-NE in Enhancing Skin Hydration Capacity

**Figure 8:**
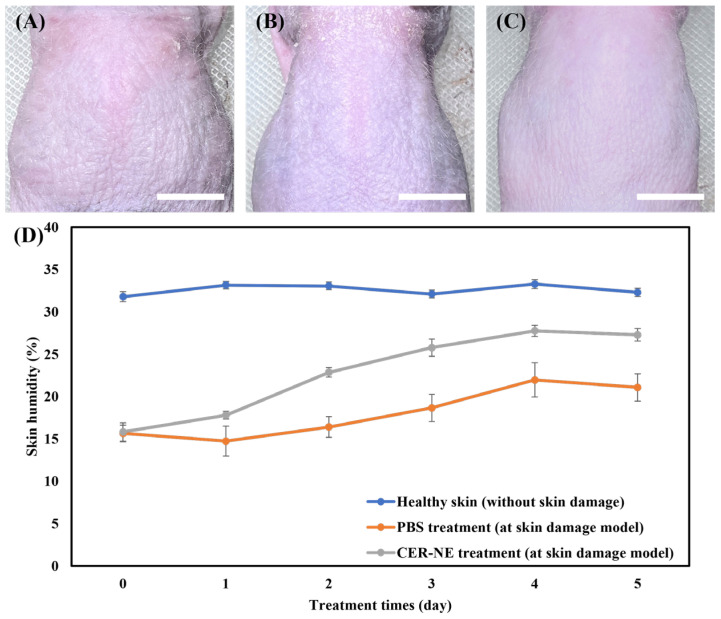
In vivo efficacy evaluation of O/W-NE after transdermal application. Skin surface morphology for (**A**) skin damage model, (**B**) PBS treatment at skin damage model, and (**C**) ceramide C2-loaded O/W-NE (CER-NE) treatment at skin damage model, (Bars: 1 cm, *n* = 2), (**D**) changes in skin humidity with treatment time. (Bars: S.D., *n* = 2).

Figure 8A–C shows the morphological images of mouse dorsal skin under a dry and damaged skin model, and treated with PBS and ceramide C2-loaded O/W-NE via transdermal delivery. Under PBS treatment, the skin exhibited dryness with visible scaling. In contrast, the skin treated with ceramide C2-loaded O/W-NE showed no such conditions, displaying elasticity and a smooth texture. Figure 8D shows the changes in skin hydration levels under different treatment conditions, demonstrating that ceramide C2-loaded O/W-NE considerably enhances skin hydration and effectively alleviates dryness, reaching approximately 186% of the untreated water content after five days of treatment. Notably, the skin hydration levels under the ceramide C2-loaded O/W-NE treatment approach those of healthy skin (without skin damage or any treatment), typically ranging from 30 to 35%. According to a previous study, delivery of ceramide to a skin injury model restored the skin barrier function to 181% of that in the untreated group after 10 days of treatment [71]. O/W-NE can transport ceramide to the epidermal layer and effectively repair the skin barrier.

## 4. Conclusions

In this study, an O/W-NE was successfully developed and optimized for the topical transdermal delivery of ceramide C2 to treat skin barrier-related disorders. The optimized formulation exhibited a mean particle size of approximately 100 nm, a narrow size distribution (PDI < 0.3), and an encapsulation efficiency of 85%, with good skin permeation and long-term application stability under refrigeration conditions. Mechanistic and in vivo studies confirmed that particle size and glycerin content play key roles in enhancing transdermal delivery without disrupting the skin barrier. Additionally, the nanoemulsion improved skin hydration in a damaged skin model, reaching approximately twice that of the untreated water content after five days of treatment, highlighting its therapeutic potential. Overall, O/W-NEs offer a safe, stable, and effective platform for the noninvasive delivery of lipophilic compounds in dermatological applications.

## Figures and Tables

**Table 1:** Optimization formulation of O/W-NE.

Items	Content
Oil and aqueous phases at volume ratios	1:9, 2:8, 3:7, 4:6
Surfactant concentration	25, 50, 75, 100, 150 mg/mL
Glycerin and deionized water at volume ratios	1:7, 1:3, 1:1, 3:1, 7:1
Ultrasonic temperature	25, 40, 60, 80 °C
Ultrasonic time	5, 10, 15, 20 min

**Table 2:** Physicochemical properties of O/W-NE.

Sample	Particle Diameter	PDI	Zeta Potential
O/W-NE	112.5 ± 1.5 nm	0.094 ± 0.013	−17.0 ± 1.8 mV
Ceramide C2-loaded O/W-NE	117.3 ± 0.6 nm	0.098 ± 0.015	−17.6 ± 1.2 mV

## Data Availability

The original contributions presented in this study are included in the article. Further inquiries can be directed to the corresponding author(s).

## Abbreviations

The following abbreviations are used in this manuscript:

O/W-NE	Oil-in-water nanoemulsion
TEWL	Trans-epidermal water loss
ICR	Institute of Cancer Research
HR-1	Hairless-1
PDI	Polydispersity index
TEM	Transmission electron microscopy
EE	Encapsulation efficiency
CLSM	Confocal laser scanning microscopy
H&E	Hematoxylin and eosin
S.D.	Standard deviation
PBS	Phosphate buffered saline
